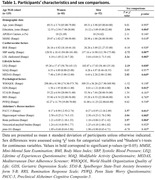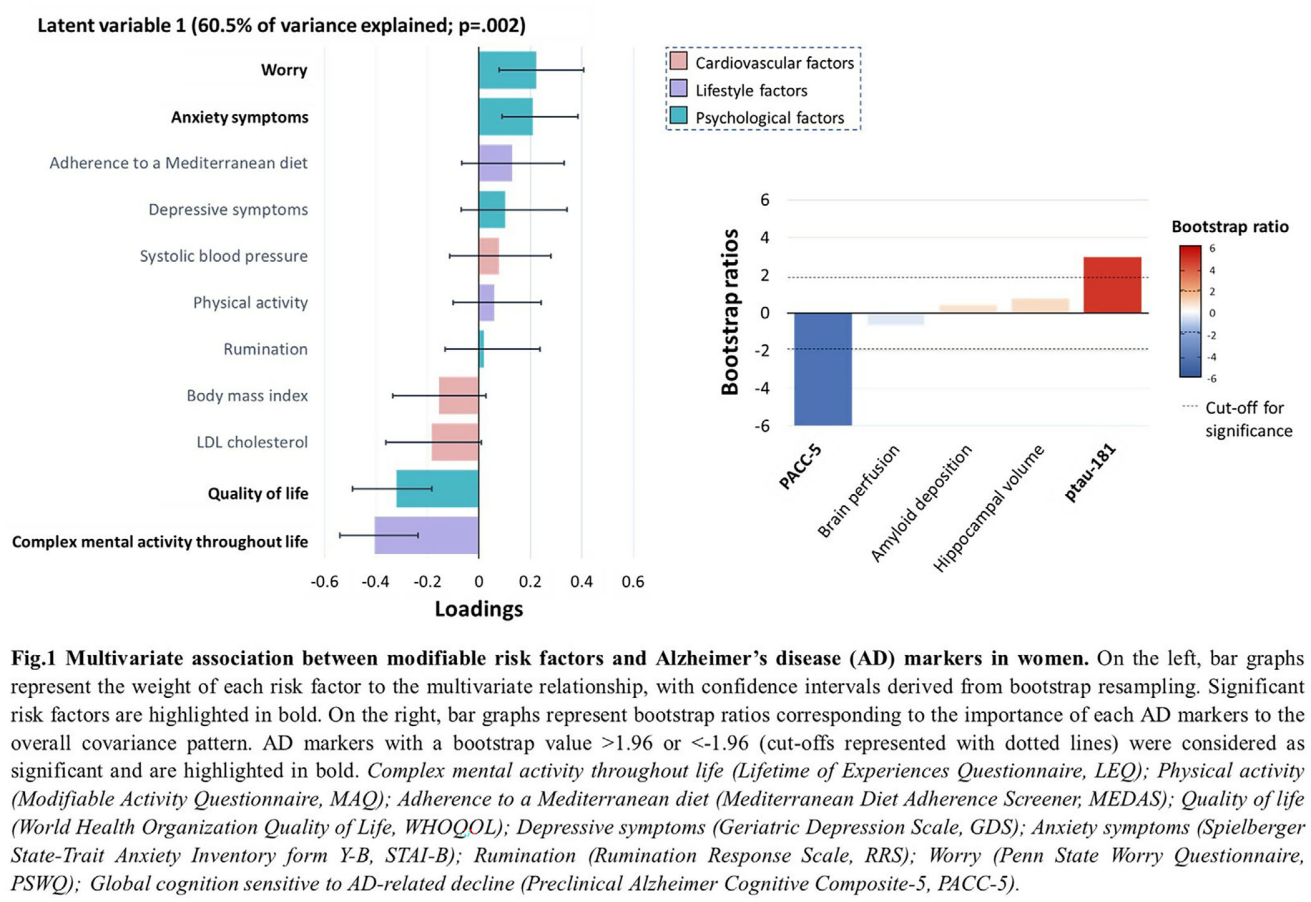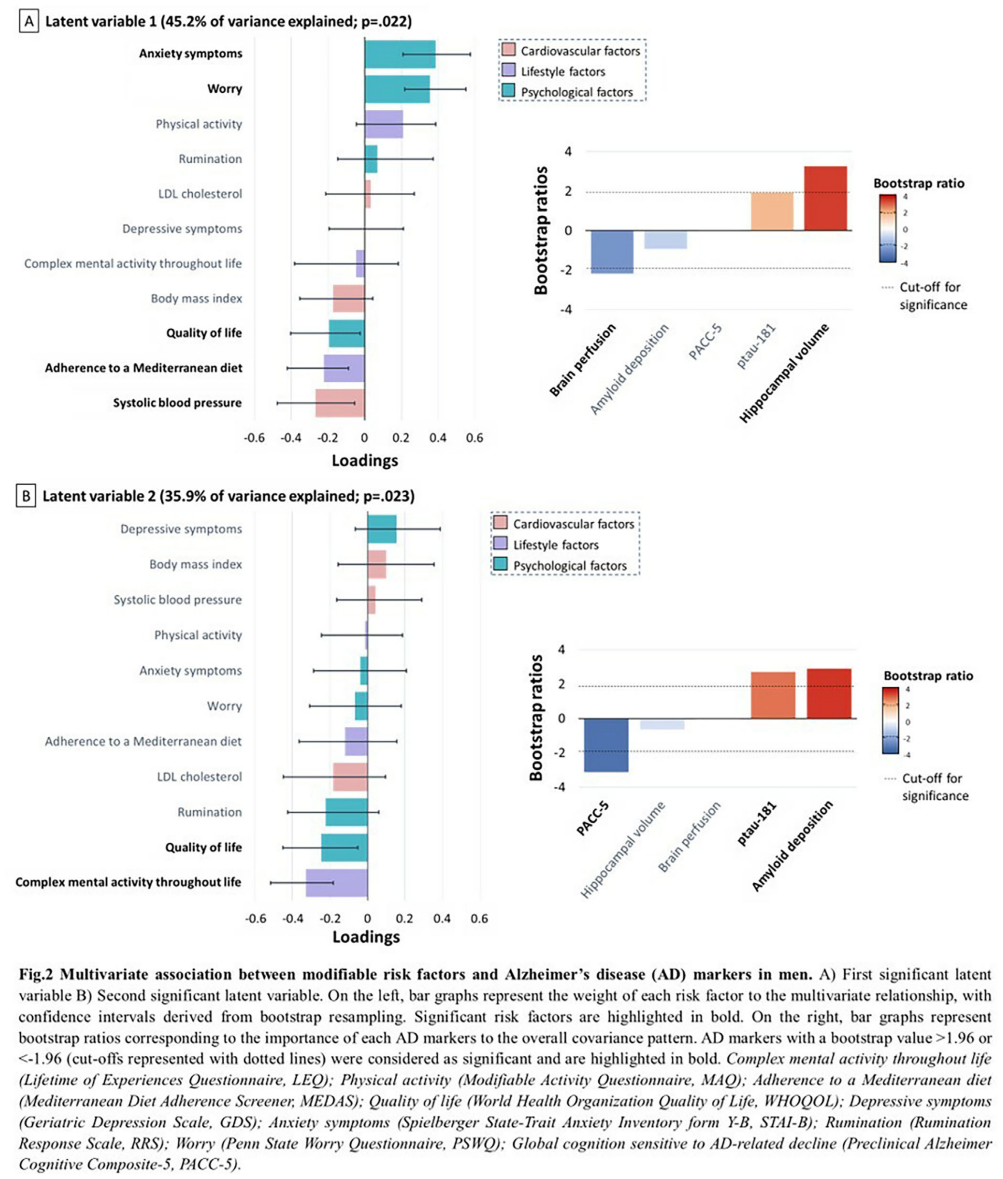# Sex‐specific risk profiles associated with Alzheimer’s disease markers in cognitively unimpaired older adults

**DOI:** 10.1002/alz.093631

**Published:** 2025-01-09

**Authors:** Edelweiss Touron, Miranka Wirth, Alexa Pichet Binette, Harriet Demnitz‐King, Fabienne Collette, Géraldine Poisnel, Denis Vivien, Vincent De la Sayette, Natalie L Marchant, Gael Chételat, Julie Gonneaud

**Affiliations:** ^1^ Normandie Univ, UNICAEN, INSERM, U1237, PhIND Physiopathology and Imaging of Neurological Disorders, NeuroPresage Team, GIP Cyceron, Caen France; ^2^ German Center for Neurodegenerative Diseases (DZNE), Dresden Germany; ^3^ Clinical Memory Research Unit, Department of Clinical Sciences, Lund University, Lund Sweden; ^4^ Division of Psychiatry, University College London, London United Kingdom; ^5^ GIGA‐CRC, University of Liège, Liège Belgium; ^6^ Service de Neurologie, CHU Caen‐Normandie, Caen France

## Abstract

**Background:**

Despite evidence that sex can modulate Alzheimer’s disease (AD) risk, whether risk factors are similarly related to AD markers in women and men remains largely unexplored. We aimed to assess how a combination of potentially modifiable risk factors are associated with cognitive and pathological markers of AD in older women and men.

**Method:**

We included 135 cognitively unimpaired older adults ( = 65 years old, 83 women; Table 1) from the Age‐Well trial (NCT02977819; baseline data) with multidomain assessments of modifiable risk factors, including cardiovascular (body mass index, systolic blood pressure, LDL cholesterol), lifestyle (complex mental activity throughout life, physical activity, diet), and psychological (quality of life, depressive and anxiety symptoms, rumination, worry). AD markers included the Preclinical Alzheimer Cognitive Composite‐5 (PACC‐5), a measure of global cognition sensitive to AD‐related decline, and multimodal neuroimaging and blood sampling providing measures of hippocampal volume (MRI), brain perfusion in temporo‐parietal regions, neocortical amyloid burden (PET) and p‐tau181 (plasma). Multivariate partial least squares analyses were used to assess relationships between combinations of risk factors and AD hallmarks (cognition, neurodegeneration, amyloid and tau) in women and men separately.

**Result:**

In women, a combination of lower quality of life and complex mental activity throughout life and higher levels of anxiety and worry, was associated with lower PACC‐5 scores and higher p‐tau181 levels (p = .002, 60.5% of variance explained; Fig.1). In men, two significant latent variables emerged. Lower systolic blood pressure, adherence to a Mediterranean diet and quality of life, and higher levels of anxiety and worry, were associated with higher hippocampal volume and lower brain perfusion in regions sensitive to AD (p = .022, 45.2% of the variance explained; Fig.2A); while lower levels of quality of life and complex mental activity throughout life were associated with worse PACC‐5 scores and higher neocortical amyloid deposition and p‐tau181 levels (p = .023, 35.9% of the variance explained; Fig.2B).

**Conclusion:**

These preliminary findings suggest that the modifiable factors that could influence AD markers may differ by sex in cognitively unimpaired older adults. Pending replication in larger and independent cohorts, these results highlight the need to consider sex specificities in prevention strategies.